# Development of artificial intelligence models for well groundwater quality simulation: Different modeling scenarios

**DOI:** 10.1371/journal.pone.0251510

**Published:** 2021-05-27

**Authors:** Naser Shiri, Jalal Shiri, Zaher Mundher Yaseen, Sungwon Kim, Il-Moon Chung, Vahid Nourani, Mohammad Zounemat-Kermani

**Affiliations:** 1 Faculty of Civil Engineering, University of Tabriz, Tabriz, Iran; 2 Water Engineering Department, Faculty of Agriculture, University of Tabriz, Tabriz, Iran; 3 Center of Excellence in Hydroinformatics, Faculty of Civil Engineering, University of Tabriz, Tabriz, Iran; 4 School of Civil Engineering, Faculty of Engineering, Universiti Teknologi Malaysia (UTM), Johor Bahru, Malaysia; 5 Department of Railroad Construction and Safety Engineering, Dongyang University, Yeongju, South Korea; 6 Department of Land, Water and Environment Research, Korea Institute of Civil Engineering and Building Technology, Goyang, South Korea; 7 Faculty of Civil and Environmental Engineering, Near East University, Near East Boulevard, Nicosia, N. Cyprus, via Mersin 10, Turkey; 8 Water Engineering Department, Shahid Bahonar University of Kerman, Kerman, Iran; Soil and Water Resources Institute ELGO-DIMITRA, GREECE

## Abstract

Groundwater is one of the most important freshwater resources, especially in arid and semi-arid regions where the annual amounts of precipitation are small with frequent drought durations. Information on qualitative parameters of these valuable resources is very crucial as it might affect its applicability from agricultural, drinking, and industrial aspects. Although geo-statistics methods can provide insight about spatial distribution of quality factors, applications of advanced artificial intelligence (AI) models can contribute to produce more accurate results as robust alternative for such a complex geo-science problem. The present research investigates the capacity of several types of AI models for modeling four key water quality variables namely electrical conductivity (EC), sodium adsorption ratio (SAR), total dissolved solid (TDS) and Sulfate (SO_4_) using dataset obtained from 90 wells in Tabriz Plain, Iran; assessed by k-fold testing. Two different modeling scenarios were established to make simulations using other quality parameters and the geographical information. The obtained results confirmed the capabilities of the AI models for modeling the well groundwater quality variables. Among all the applied AI models, the developed hybrid support vector machine-firefly algorithm (SVM-FFA) model achieved the best predictability performance for both investigated scenarios. The introduced computer aid methodology provided a reliable technology for groundwater monitoring and assessment.

## 1. Introduction

Humans depend mainly on groundwater for both drinking, agricultural, and industrial purposes [1, 2]. It is, therefore, necessary to perfectly understand the geochemical processes that regulate the chemical composition of groundwater as it will improve the understanding of the hydro chemical systems in different regions around the world [3]. Such information can also improve groundwater resource management and utilization by highlighting the relationships between groundwater quality, aquifer lithology, and recharge type [4, 5]. With the traditional approaches of water resource management, surface water and groundwater systems are considered as two separate entities; however, both systems have been proven to affect each other from both qualitative and quantitative perspective based on the recent development in land and water resources analysis [6, 7]. Nevertheless, groundwater contamination, either by anthropogenic activities, or by inherent aquifer material composition, reduces groundwater supply capacity or restricts its exploitation [8, 9]. The quality of groundwater could also be influenced by agricultural activities, such as the use of fertilizers and pesticides even though other geological and anthropogenic activities can also influence groundwater quality [10, 11] since it is a component of physical and chemical parameters that is affected by human and geological activities [12–14].

Normally, the traditional approach to groundwater quality analysis depends mainly on mathematical modeling such as time series analysis, probability statistics, etc. These methods assume the existence of a linear relationship between the dependent and independent variables; hence, the overall accuracy of such models is usually low [15, 16]. Considering the prevailing issues in simulating groundwater quality [17, 18], there is a need for new computational approaches to this problem. The advancement of AI models in the field of hydrology and environment has received a massive attention over the past decade [19–23]. In this regard, some studies have been focused on the development of some computational approaches to groundwater quality simulation; for instance, Yesilnacar et al. [24] developed an artificial neural network (ANN) model for the prediction of groundwater nitrate concentration in Harran Plain in Turkey. The study reported that the developed model succeeded in achieving a cost-effective management of groundwater resources. Furthermore, Liu et al. [25] tested support vector machine (SVM) model that relies on eight assessment indicators for water quality assessment. It was determined that the proposed SVM model performed well in determining water quality grade as recommended by the Groundwater Quality Assessment Standard. The proposed method also succeeded in solving complex nonlinear relationships that exists between water quality grade and assessment factor; the model also achieved a high level of prediction accuracy and provided a feasible and reasonable performance as an assessment method. An ANN model has been developed by Yesilnacar and Sahinkaya [13] for groundwater sulfate (SO_4_) and Sodium Adsorption Ratio (SAR) concentration prediction. The outcome of the study revealed the possibility of managing groundwater resources in an easier and cost-effective manner with the proposed model. A Bayesian neural network (BNN) model has been developed by Maiti et al. [26] for evaluation of groundwater quality. The study also proved that the model could provide useful constrain (based on uncertainty and statistical analyses) that could be useful in assessing and monitoring the quality of groundwater.

The evaluation of the quality of surface and ground water in rural areas of the Silesian Lowlands has been presented by Orzepowski et al. [27], under variable climatic conditions. The outcome of the study showed that the ANN modeling method based on statistical analysis served as a useful tool for water content estimation in soils under various climatic conditions. The study by Khaki et al. [28] employed Adaptive Neuro-fuzzy Inference System (ANFIS) and ANN for the simulation of total dissolved solids (TDS) and electrical conductivity (EC) levels. Both techniques demonstrated effectiveness in interpreting the behavior of water quality parameters. The potential of ANN model in predicting SAR, magnesium absorption ratio, residual sodium carbonate, Kellys ratio and percent sodium (%Na) in groundwater has been evaluated by Wagh et al. [29]. The outcome of the study proved the effectiveness of the developed ANN model in making accurate prediction that contribute to the irrigation suitability indices. Barzegar and Moghaddam [30] comparted the performance of three ANN algorithms including Multi-Layer Perceptron (MLP), radial basis function neural network (RBFNN) and generalized regression neural network (GRNN) in predicting the salinity of groundwater as expressed by the electrical conductivity [EC (μS/cm)]. From the modeling results, the three models performed well in predicting salinity of groundwater. The capability of SVM model for nitrate concentration prediction has been evaluated by Arabgol et al. [31] in Arak plain groundwater, Iran. The SVM model succeeded in predicting the nitrate concentration based on a set of groundwater quality variables that could be easily measured, such as the water temperature, groundwater depth, electrical conductivity, dissolved oxygen, pH, total dissolved solids, land use, and season of the year. The study also showed that the SVM model is a fast, reliable, and cost-effective AI technique. The feasibility of AI techniques in groundwater quality simulation has been evaluated by numerous scholars and such studies have produced efficient performances [32–40].

Gholami et al. [34] presented an advanced form of the ANFIS model for groundwater quality simulation; the proposed model was described as coactive-ANFIS (CANFIS) integrated with geographic information system (GIS). The training and validation of the proposed model was performed by considering a case study of Mazandaran Plain in the northern region of Iran. The outcome of the study demonstrated the efficiency of incorporating AI model with GIS. The study by Azimi et al. [41] presented ANN and modified fuzzy clustering models for the evaluation of decreases in the quality of drinking water. The performance of the models was evaluated on real instances of the southeast aquifers in the central region of Iran. The study reported the capability of the modified clustering method to improve the prediction efficiency of the model when compared to the previous reports. The feasibility of ANN and multiple linear regression (MLR) models in modeling the Canadian Water Quality Index (CWQI) of groundwater has been evaluated by Nathan et al. [42]; the study optimized the input modeling parameters using Hierarchical Cluster Analysis (HCA) approach and clustering procedure. From the analysis of the results, both MLR and ANN models were found as reliable methods of predicting the CWQI. The study further indicated that the research finding could assist decision makers in addressing water quality-related problems.

Furthermore, efforts are still dedicated to the development of novel AI predictive models that could reliably handle the diversity, non-linearity, and non-stationarity of the groundwater quality pattern. The study by Barzegar et al. [43] investigated the feasibility of using extreme learning machine (ELM) model as a new and advanced version of ANN model for the prediction of the level of fluoride contamination in groundwater. Upon validation against the classical AI models, the ELM was proven capable of predicting the level of fluoride contamination. Different studies have tried using hybridized AI models for groundwater quality simulation; among the studied AI models are the nature-inspired optimization algorithms like particle swarm optimization, differential evolution, genetic algorithm, ant colony algorithm, firefly algorithm, etc. [44–47].

The study by Sepahvand et al. [48] focused on the performance of four AI models in predicting SAR; the evaluated models are M5P model tree, RF, implementing bagging algorithm on M5P, and group method for data handling (GMDH). From the results of the study, bagging M5P model tree model achieved higher accuracy in SAR prediction compare to the rest of the models in a given study area. Another study evaluated Gaussian Process (GP), M5P, RF and random tree (RT) model for the prediction of nitrate and strontium contamination in groundwater [49]. The study showed that GP model achieved better performance compared to the other models in terms of nitrate and strontium concentrations prediction.

**Fig 1:**
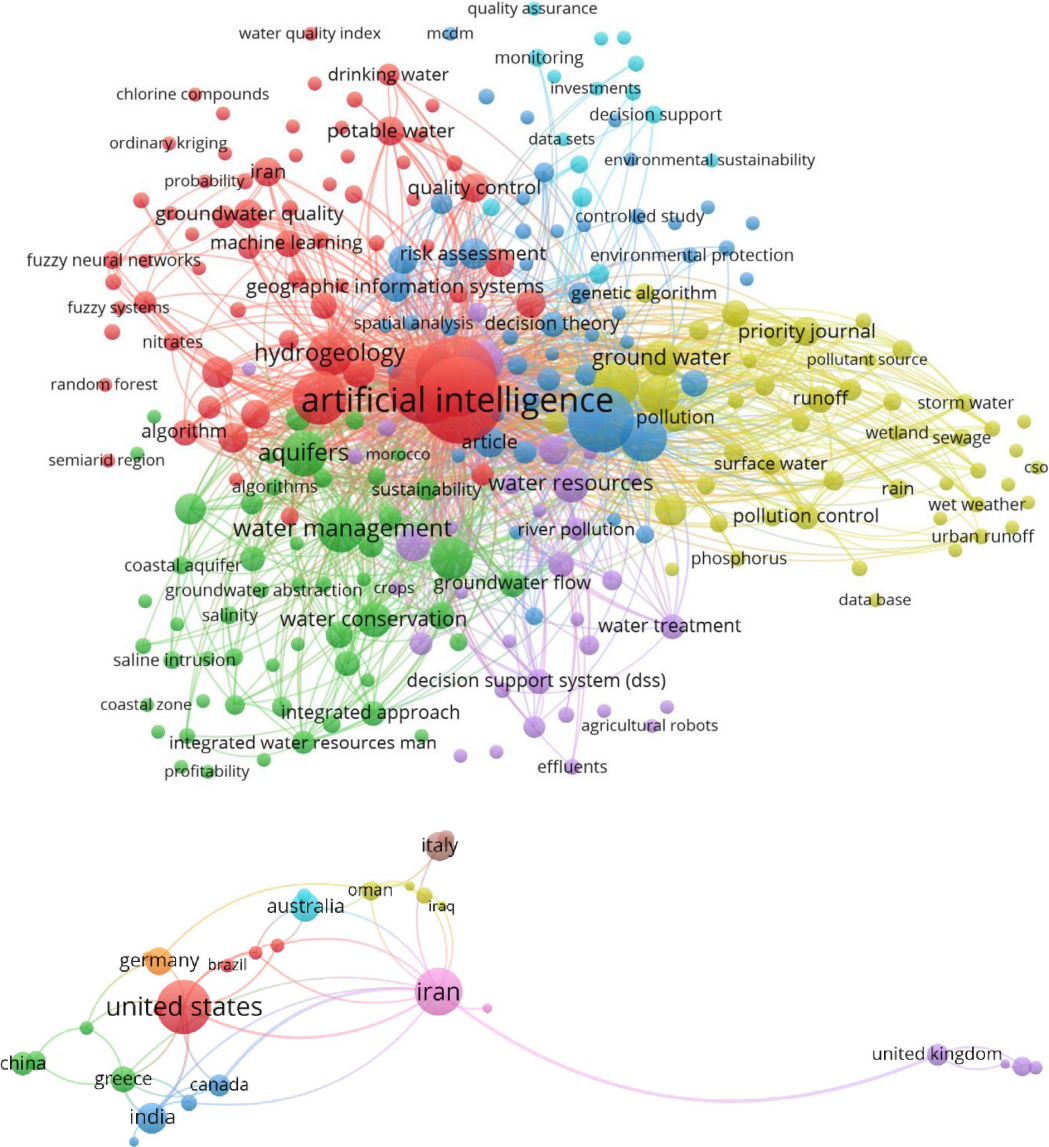
The literature review analysis for the groundwater quality simulation using the capacity of the AI models over the past two decades and the counties who conducted research on this research domain.

Owing to the need for studies on the modeling of groundwater quality for diverse geoscience engineering applications, various studies have been dedicated to this course over the past decade. Based on the research adopted in Scopus database “Keywords search: Groundwater water artificial intelligence”, the search finding indicated there are 131 research articles published in this domain covering the time period 1988–2021. Based on the literature analysis conducted on the collected database using VOSviewer, the intersection occurrence keywords are 268 with 6 clusters visualization (Fig 1). Also, the literature indicated 41 countries were established research on this research domain. Based on the results presented in Fig 1, Iran region is the second top countries after USA is focusing on the simulation of the groundwater quality. In addition, Fig 1 signified that there is no established research on the hybridized AI models in this research domain.

Although substantial studies have been performed to estimate groundwater quality parameters using soft computing methods, a comprehensive study that can evaluate the abilities of those models using both quality parameters and geographical information is still rare in literature. Hence, the present study aimed to predict four important groundwater quality parameters in Tabriz Plain, Iran using eight soft computing approaches. Tabriz Plain is located in the vicinity of Lake Urmia (the second great saline lake of the globe), which has experienced sever water level down during a couple of recent decades. Given that the groundwater table level has been decreased in the wells, it is usually assumed that there is a hydraulic interaction between the lake and surrounding aquifer (of Tabriz Plain) that may cause salt water intrusion into the aquifer. This might reduce its quality for different agricultural, domestic and industrial customers. Nonetheless, numerous agricultural and industrial activities have been carried out in this plain that can contribute to variations in water quality parameters. Therefore, having robust tools for spatially simulation of the groundwater quality parameters is of crucial importance in this region. Nevertheless, the proposed protocol might be translated to other regions where the necessary information for model feeding is available.

## 2. Materials and methods

### 2.1. Study area

**Fig 2:**
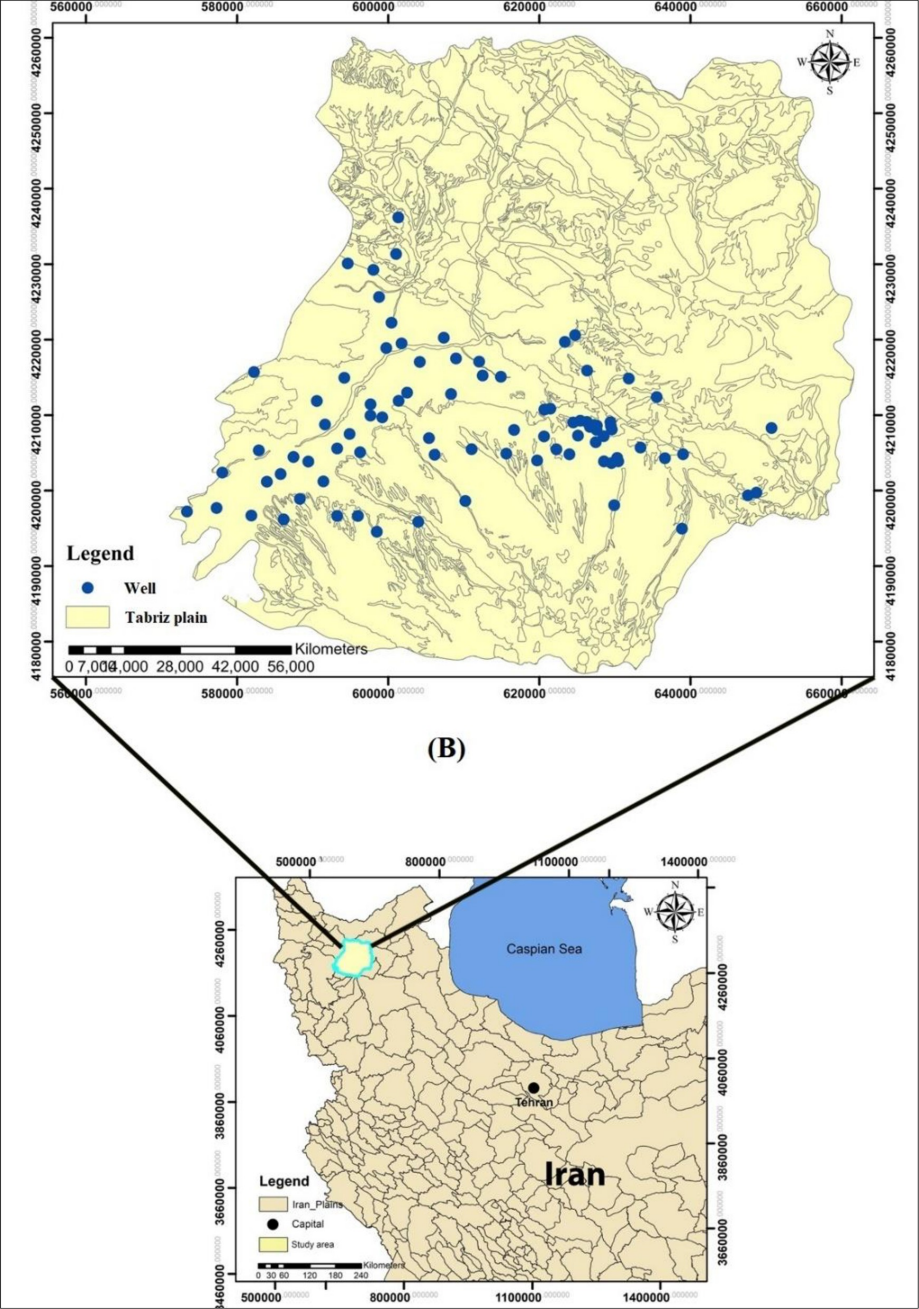
The selected case study at the Tabriz Plain, Iran.

The study area covers the Tabriz Plain in Northwestern Iran that has been located between the latitudes of 45°30′ and 46°15′ N and altitudes 37°56′ and 38°17′ E, with a total catchment area of more than 700 km^2^ [50]. Tabriz area lies in east Azerbaijan province, which is structurally part of the central Iran unit. It is wedged between the Zagros and Alborz mountain systems. The area includes formations of Devonian to Quaternary age affected by various geologic movements, most strongly those of Alpine origin. The Tabriz area formations are composed of Miocene faces that have covered alluvial sediments unevenness and have formed steep strata east to west. Miocene bedrock in this area is high, so alluvial sediment is very thin. The altitude ranges from 1247 to 3600 m.a.s.l. The average annual precipitation of the area is about 230 mm, with an average annual temperature value of 12.8 ^o^C. The climatic context of the studied area is cold and dry based on to the Emberger climate classification index [50]. Observational data from 90 wells were collected including different groundwater quality parameters. The location of the study area and Tabriz Plain presented in (Fig 2, https://www.diva-gis.org/gdata). A brief description of the utilized parameters is presented in Table 1. A brief description of the utilized parameters has been presented in Table 1. The major perennial river of the plain is Ajichai River. Data from 90 observational wells across the plain were used for evaluating the adopted methodology. The data have been received from Regional Water Company of East Azarbaijan, where the data have been thoroughly analyzed and screened for any inconsistency. The water quality records included various parameters, e.g. Ca, Mg, Cl, SO_4_, EC, etc have been measured during a 15 years period (monthly records are available between 2005–2019).

**Table 1:** Statistical characteristics of the utilized groundwater quality parameters.

Parameter	min	max	mean	SD	C_V_	C_SX_
**EC (mmhos/cm)**	267.25	11261.33	2322.006	2224.309	0.957	1.485
**SAR (-)**	0.668	27.174	6.416	5.587	0.870	1.823
**SO**_**4**_ **(meq/l)**	0.227	12.632	4.048	3.110	0.768	0.702
**TDS (mg/l)**	163.92	6880.727	1475.033	1423.711	0.965	1.407
**Ca (meq/l)**	1.054	28.466	5.949	5.664	0.952	2.191
**Mg (meq/l)**	0.595	15.733	4.774	3.968	0.831	0.920
**Na (meq/l)**	0.606	81.423	11.966	15.069	1.259	2.025
**Cl (meq/l)**	0.29	91.156	14.178	18.777	1.324	1.770
**TH (mg/l)**	99.25	2087.964	511.593	432.098	0.844	1.518
**K (meq/l)**	0.425	0.545	0.212	0.119	0.563	0.874

Note: C_V_ = coefficient of variation; C_SX_ = Skewness coefficient

### 2.2. Artificial intelligence models

In this study, several types of AI models are used to predict groundwater quality parameters. It is worth noting that in all of the applied models, prior to entering the input data, the dataset has been standardized and confined in the [0,1] range. The applied models can be categorized as i) network-based models (Artificial Neural Network, ANN, and Adaptive Neuro-fuzzy Inference System, ANFIS), ii) classification-based analysis (Support Vector Machine, SVM), iii) regression-based analysis (Multivariate Adaptive Spline Regression, MARS), iv) ensemble tree-based technique (Random Forests, RF), and v) integrative models (embedded Firefly Algorithm with ANN and SVM, ANN-FA and SVM-FA). In the following, a concise description of these models is provided.

#### 2.2.1. Multi-Layer Perceptron neural networks

**Fig 3:**
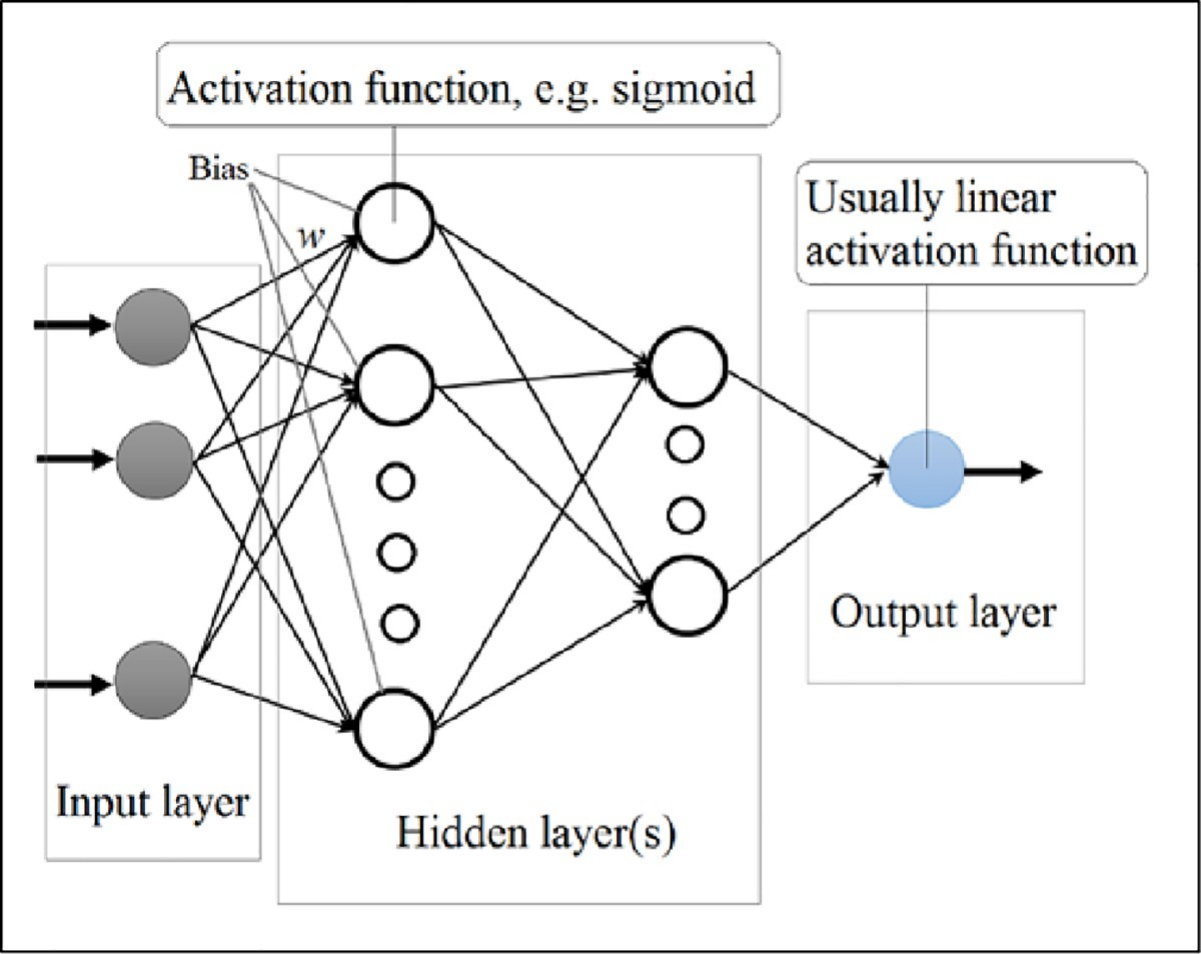
Schematic structure of a four-layer MLP neural network.

Multi-Layer Perceptron (MLP) neural networks are basic types of Feed Forward Neural Networks (FFNN), which are parallel layered structure networks. In an FFNN, the network calculation flows forward from the first layer to the last one. The layers in an MLP network are fully connected with the previous and the next layer. Normally, an MLP network consists of three layers, namely the input layer, the hidden layer, and the output layer. The input layer gets the input parameters and acts as an entrance to the network. The calculation process in the hidden and output layers is based on several interconnected processors called neurons (see Fig 3). In other words, in a typical MLP model, each neuron in a layer receives information from all the neurons in the previous layer and accordingly dispatches information to the neurons in the next layer. The calculated information moves based on the synaptic weights and biases in the network. In the MLP, two functions − summation and activation functions − are used to transmit the input calculated information of a neuron and prepare it to be sent to the neurons in the next layer. Eq (1) shows how the summation function (*S*_*j*_) of the *j*^*th*^ neuron acts by receiving the input variables (*I*_*i*_) to a specific neuron.

$$S_{j}=\sum_{i=1}^{n}w_{ij}I_{i}+\beta_{j}$$ (1)

In Eq (1), *n* denotes the total number of inputs, *w*_*ij*_ are the connection (synaptic) weights, and *β*_*j*_ shows the bias value. Having the outcome of Eq (1), the activation function (e.g., the sigmoid activation function, $f_{j}(x)=1/(1+e^{-S_{j}})$ for the hidden layer, and linear function for the output layer, *f*_*j*_(*x*) = *S*_*j*_) calculates the output value of the neuron. In the end, the final output is attained based on Eq (2):

$$y_{i}=f_{i}\left(\sum_{i=1}^{n}w_{ij}I_{i}+\beta_{j}\right)$$ (2)

After constructing the structure of the network, the adjustable parameters, including the connection weights and biases, should be tuned. Hence, during the training phase of the network, a learning algorithm such as gradient descent algorithm is used to update the adjustable parameters. Detailed information about the MLP networks can be found in several studies [51–53]. In this work, we used the following parameters for forming the final architecture of the MLPNN: the number of hidden layers = 2, number of hidden nodes = 17, activation function for the hidden layer nodes = tangent hyperbolic; activation function for the output layer node = tangent hyperbolic.

#### 2.2.2. Support Vector Machine (SVM)

Derived from the statistical learning theory, the SVM has attained a lot of attention during the last decades in simulating engineering problems. Similar to the MLP network, the SVR, which is the regressive version of the support vector machine (SVM), is considered as a supervised machine learning algorithm; however, the methodology applied is different. In order to construct an SVM, the data is separated into several regressive subclasses using a decision surface so-called a hyperplane. The hyperplane transforms the nonlinear input space to a high dimensional area [54].

Each class has similar features, which make the SVM capable of capturing the nonlinearities of a complex system and extend it to predict response values. Those marginal data which are close to the hyperplane form support vectors. The support vectors are critical elements for training the SVM model [55].

By extending the SVM method for simulating and predicting problems, the SVR network can be introduced as below [56]:

$$f(x)=\sum_{i=1}^{n}\alpha_{i}k(x,x_{i})+\beta$$ (3)

where *x* is the input dataset and *n* is the number of input data. *α* and *β* are the Lagrange multiplier and bias, respectively. *k*() denotes the kernel function.

The performance of an SVM highly depends on the utilized kernel function. There are several available kernel functions that might be used in an SVR; however, four types of them are more common than the others, such as the linear, polynomial, radial basis function (RBF), and sigmoid kernel functions [57, 58]. In this research, the RBF kernel function is applied for creating the SVM models.

$$k(x_{i},x)=\exp\left(\gamma {\left\|x_{i}-x\right\|}^{2}\right)$$ (4)

where *γ* represses the width value of the RBF kernel.

#### 2.2.3. Integrative firefly machine learning models (ANN-FA and SVM-FA)

Using metaheuristic algorithms, such as the Genetic Algorithm (GA) algorithms, is an alternative approach for training the machine learning models i.e., ANN and SVM. In this research, the Firefly Algorithm (FA) is embedded in the MLP and SVM models to create the integrative models of MLP-FA and SVM-FA. The FA algorithm is inspired by the natural behavior of fireflies in attracting each other based on their flashing mechanism [59]. Mathematically speaking, each firefly represents a possible solution. The objective function is introduced by the light intensity of each firefly. Fireflies with lower light intensity (*x*_*j*_) follow those with higher light intensity (*x*_*i*_). The following formula shows this movement mechanism:

$$x_{i}=x_{i}+\beta (x_{j}-x_{i})+\alpha (u-0.5)$$ (5)

$$\beta =\beta_{0}[\exp\left(-\lambda .r_{ij}^{2}\right)]$$ (6)

In the above formulae, *λ* denotes the absorption coefficient. The Euclidean distance between the *i* and *j* fireflies is shown as *r*_*ij*_. *β* and *β*_0_ define the firefly’s attractiveness and maximum possible attractiveness. U is a random number within the range of null and unity, and *α* represents the random movement of the fireflies, which is named as the trade-off constant. Following the above movement mechanism, all the fireflies move toward the firefly with the highest light intensity (best firefly). The best firefly explores the search space randomly [60].

In this study, the FA algorithm is employed to update the tuning parameters of the standard SVM model, including the hyperplane parameters, as well as weights and biased in the MLP neural network [61]. By using this algorithm, the attraction coefficient was set as 1, light absorption coefficient as 0.4 and a cooling factor of 0.9.

#### 2.2.4. Multivariate Adaptive Regression Splines (MARS)

The MARS model is a data-driven model based on the concepts of forward and backward stepwise regression analysis. At the first step (forward part), a suitable set of explanatory variables is selected. Afterward, with a combination of the selected variables and presenting the location of knots, some linear functions are constructed in the solution space [62].

At the backward procedure, the unnecessary variables are removed, which have been previously selected at the forward step [63]. Hence, the variable X would be updated to the variable Y according to one of the following relations:

Y=max(0,X−c) (7)

Y=max(0,c−X) (8)

c is known as the threshold value.

#### 2.2.5. Random Forests (RF)

**Fig 4:**
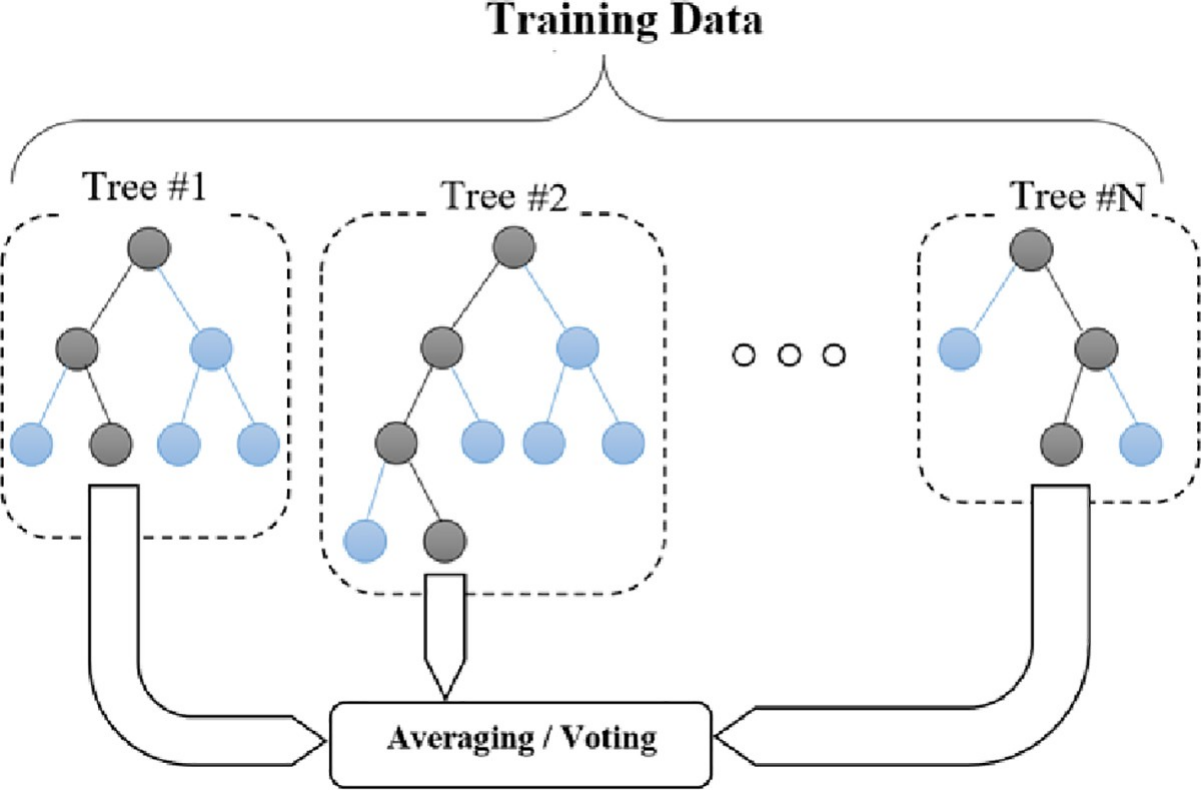
The schematic architecture of an RF model.

The RF consist of many decision/regression trees (as weak learners) which uses an extended version of the bagging technique to create a strong ensemble model. The trees are built on random subsets (different samples) of data. Thus, equivalent to the number of training samples, several decision/regression trees grow [64]. After growing the trees, the final output is calculated according to the ensemble technique i.e., voting for the classification and averaging for the regression problems (Fig 4). Generally, in the RF model, three types of tuning parameters should be determined, such as the number of trees (here it ranged between 100 to 150), the maximum depth of the trees, and the number of selecting features (here, 6 for the first scenario and 2 for the second scenario) upon every split [65].

#### 2.2.6. Adaptive Neuro-Fuzzy Inference System (ANFIS-GP and ANFIS-SC)

ANFIS are simulative/predictive machine learning systems that are composed of ANNs, as the network structure of the ANFIS, and a Takagi-Sugeno Fuzzy Inference System (FIS), as the fuzzy logic theory (fuzzification and defuzzification procedures) of the model. In FIS, several fuzzy rules are produced, which allow for an appropriate study of a complex and nonlinear system. The neural network structure of the ANFIS updates the tuning parameters of the FIS i.e., the geometric parameters of the introduced Membership Functions (MFs) [66]. The ANIFS architecture involves five layers, including the fuzzification layer (the first layer), the rule layer (the second layer), the normalization layer (the third layer), the defuzzification layer (the fourth layer), and finally the output layer.

Developing an ANFIS model takes two major steps. At the first step, namely the constructing step, the general architecture of the network, i.e., the number and the types of the MFs and the rule-based FIS, is determined. In this study we used trapezoidal shape membership functions to construct 3 numbers of rules. The second step refers to the training phase of the ANIFS. The learning procedure of the ANIFS is a hybrid method that uses the back-propagation technique for the nonlinear premise parameters (the parameters of MFs) and the least square error for the linear consequent parameters (coefficients of the fuzzy if-then rules). It should be mentioned that in this study, two different approaches, such as General Partitioning and (GP) and Subtractive Clustering (SC) techniques, are employed to construct the ANFIS models (ANFIS-GP and ANIFS-SC). Detailed information regarding these models has been given in Benmouiza and Cheknane and Wang et al. [67, 68].

### 2.3. Modeling flowchart

Two modeling scenarios were adopted here for modeling the target parameters:

1. First scenario: Different groundwater quality parameters (Ca, Mg, Na, Cl, total hardness and K) were applied as input parameters for simulating the target parameters, e.g., total dissolved solids (TDS), electrical conductivity (EC), Sulphate (SO_4_) and sodium adsorption ratio (SAR). The input variables were selected on the basis of correlation analysis as well as chemical judgment.
2. Second scenario: The geographical coordinates of the studied wells were used as input variables to simulate the target parameters. Hence, instead of the influential parameters on each target parameter, the locations coordinates were introduced, and the models simulated the spatial distribution of the targets based on this information. Application of soft computing models with this scenario is similar to the geo-statistics approaches, where the spatial coordinates are used for interpolating the values of parameters in any specified region. This would be very important step forward, because ground quality parameters can be estimated in regional scale by having only coordinate information. This will be exclusively important for the regions with data scarcity.

With any adopted scenario, a major task with applying soft computing models is partitioning the available patterns into training and testing data. Here, a k-fold testing type data management strategy was adopted, and the models were trained each time using “k-1” blocks of data and then tested using the remaining one block patterns. The process was repeated until all available patterns were participated in both the training and testing stages. A simpler way might be dividing all patterns into 2 blocks as it is common in such kinds of studies. However, such simple data assignment does not allow a through scanning of all the available patterns, so the obtained results would be partially valid. Hence, applying k-fold testing would provide enough detailed information about the models’ capability/stability in mapping the nonlinear relationships between the input and target variables. Using the k-fold testing strategy (with k = 1), all available patterns were divided into 90 blocks (each block contained all available patterns of a single well) and each model was trained and tested 90 times for simulating each parameter. With each soft computing model, the process was repeated 90 times for each target parameter, so total 90 training-testing procedures were conducted for simulating each target parameter by each model. Hence, a total 360 training-testing processes were fulfilled for simulating 4 target parameters in the first scenario. In case of the second scenario, similar protocol was adopted, where the k-fold testing strategy (with k = 1) was used for assessing the models performance. Hence, the total available patterns of a single well were reserved each time as testing patterns, while the models were trained using the total patterns of the rest of the wells (89 wells). The process was repeated until the data from all the wells participated in training-testing stages. With the second scenario, total 360 training-testing processes were established, too.

### 2.4. Evaluation criteria

Three statistical evaluation criteria, namely, the determination coefficient (R^*2*^), the scatter index (*SI*) and the Nash-Sutcliffe coefficient (*NS*) were used to assess the employed models [69]:

$$SI=\frac{RMSE}{\bar{x}}=\frac{\sqrt{\frac{1}{n}{\sum_{i=1}^{n}\left(x_{io}-y_{io}\right)}^{2}}}{\bar{x}}$$ (9)

$$NS=1-\frac{\sum_{i=1}^{n}(x_{io}-y_{io}{)}^{2}}{\sum_{i=1}^{n}(x_{io}-\bar{x}{)}^{2}}$$ (10)

where, *x*_*io*_ and *y*_*io*_ are the observed and estimated values of each parameter at the *i*^th^ time step, respectively. $\bar{x}$ stands for the mean observed values and *n* is the number of available patterns (locations). *NS* is an indicator of analyzing the variance of the simulated and observed values, where *NS* = 1 shows the perfect fit. For the adopted data management strategy, the applied indices were computed per test stage as well as for the all available patterns.

## 3. Results and discussion

The present paper aimed at modeling four important groundwater quality parameters, e.g., EC, SAR, TDS and SO_4_ values through employing eight different soft computing techniques with two modeling scenarios. In the next subsections, the overall statistical analysis of the model’s performance accuracy is presented for both the adopted scenarios.

### 3.1. First modeling scenario results

Table 2 sums up the global statistical indices of the applied models that have been computed by making a global simulation vector of the complete data set (data from all wells for the test period). For obtaining these results for the first scenario, a global matrix was built at each well comprising the observed and corresponding simulated values of target parameters of all test stage. Then, the statistical indicators were computed for each well. Finally, the global indicators were obtained through averaging the indicators of all studied wells. For the second scenario, as the test patterns belonged to a single way at each k-fold testing stage, average values of the indicators, which have been computed for each well, were computed and reported as global indicator values.

**Table 2:** Global statistical indices of the applied predictive models.

	**EC**			**SAR**			**TDS**			**SO**_**4**_		
***First modeling scenario***												
	***R***^***2***^	***SI***	***NS***	***R***^***2***^	***SI***	***NS***	*R*^*2*^	*SI*	*NS*	*R*^*2*^	*SI*	*NS*
MARS	0.996	0.139	0.978	0.915	0.251	0.915	0.999	0.019	0.999	0.946	0.178	0.945
RF	0.974	0.153	0.973	0.952	0.234	0.926	0.901	0.425	0.803	0.941	0.185	0.940
ANFIS-GP	0.985	0.226	0.943	0.906	0.264	0.906	0.999	0.026	0.999	0.846	0.302	0.843
ANFIS-SC	0.986	0.203	0.954	0.893	0.285	0.891	0.999	0.014	0.999	0.858	0.291	0.854
ANN	0.942	0.260	0.925	0.895	0.282	0.893	0.999	0.023	0.999	0.900	0.240	0.900
ANN-FFA	0.982	0.131	0.980	0.930	0.232	0.928	0.999	0.026	0.999	0.943	0.184	0.941
SVM	0.982	0.210	0.951	0.905	0.276	0.898	0.991	0.119	0.984	0.842	0.305	0.839
SVM-FFA	0.986	0.111	**0.986**	0.935	0.219	**0.935**	0.999	0.023	**0.999**	0.949	0.174	**0.947**
***Second modeling scenario***												
MARS	0.923	0.266	0.962	0.916	0.245	0.965	0.937	0.286	0.957	0.893	0.289	0.952
RF	0.916	0.307	0.950	0.917	0.252	0.963	0.917	0.325	0.944	0.887	0.296	0.949
ANFIS-GP	0.824	0.400	0.915	0.883	0.302	0.947	0.867	0.386	0.922	0.830	0.364	0.924
ANFIS-SC	0.903	0.308	0.950	0.905	0.276	0.956	0.901	0.326	0.944	0.891	0.301	0.948
ANN	0.749	0.485	0.876	0.872	0.342	0.932	0.833	0.402	0.915	0.815	0.372	0.920
ANN-FFA	0.910	0.297	0.953	0.938	0.211	0.972	0.933	0.249	0.967	0.933	0.262	0.960
SVM	0.890	0.316	0.947	0.890	0.294	0.950	0.880	0.364	0.930	0.864	0.339	0.934
SVM-FFA	0.949	0.226	**0.973**	0.948	0.207	**0.975**	0.939	0.239	**0.970**	0.946	0.209	**0.974**

In case of the *EC* modeling, the most accurate results belonged to the hybrid SVM-FFA model (with the lowest *SI* and the highest *NS*), followed by the ANN-FFA, MARS and RF models. However, the highest *R*^*2*^ values were observed for MARS model in comparison with the other established predictive models. This could be explained through exclusive specification of this index that captures only the linear dependency between tow set of events and can take higher values (around unity) even with higher error magnitudes. Therefore, this can’t be solely applied as a verification index of modeling performance as has been advised by Legates and McCabe (1999) [70].

The coupled SVM-FFA model has improved the performance accuracy of the SVM model by 9.9% and 3.5% reduction/increase in the *SI* and *NS* values, respectively. Similarly, ANN-FFA model has made 12.9% reduction and 5.5% increase in *SI* and *NS* values, respectively. Regarding the *SAR* simulation models, again, the SVM-FFA outperformed the rest of the models, followed by the ANN-FFA, RF and MARS models. SVM-FFA improved the performance accuracy of the SVM models by 5.7% reduction in *SI* value and 3.7% increase in *NS* value. Comparing with the *EC* simulation models, the overall performance accuracy of the models in this case was low (Δ*SI* = 0.11 for the SVM-FFA model). Attending to the TDS and SO_4_ estimation models the same observations were made where the coupled SVM-FFA surpassed the other applied models and differences between the error magnitudes of models were considerable in some cases.

Overall, it was observed that when the models relied on some groundwater quality parameters to spatially estimate the EC, SAR, TDS and SO_4_ values, the *SAR* estimation models gave less accurate results than the models established for simulating other three parameters. Although all four parameters are related to groundwater qualitative aspects, such differences might be explained through the vectors that have participated in forming these parameters. The main factors affecting the *SAR* values are Na^+^, Ca^2+^ and Mg^2+^ that are easily soluble cations in the soil (especially, the sodium solubility is very high), so they can be leached from soil horizons during the precipitation or irrigation events that deliver considerable amounts of water and make deep percolation [71]. Such high variations through the soil vertical profile might affect the modeling accuracy and make the interpolations among different locations difficult.

### 3.2. Second modeling scenario results

The statistical indexes of the models established on the basis of the geographical coordinates of the wells (second scenario) have been listed in Table 2. First, the performance accuracy of the models has been decreased when relying on geographical coordinated in comparison with the models developed on groundwater quality parameters, as could be anticipated. This might be due to the inclusion of interrelationships between the input-target parameters in the first scenario. However, the overall accuracies of the models developed using the second scenario are almost comparable and practically sound. In similarity to the previous cases, the SVM-FFA and ANN-FFA models presented their superiority over the rest of the applied models in modeling all studied parameters, while ANN provided the less accurate results. However, unlike to the previous scenario, the models simulating the *SAR* values presented the most accurate results, although as discussed, inclusion of Na^+^, Ca^2+^ and Mg^2+^ can make its interpolations difficult.

**Fig 5:**
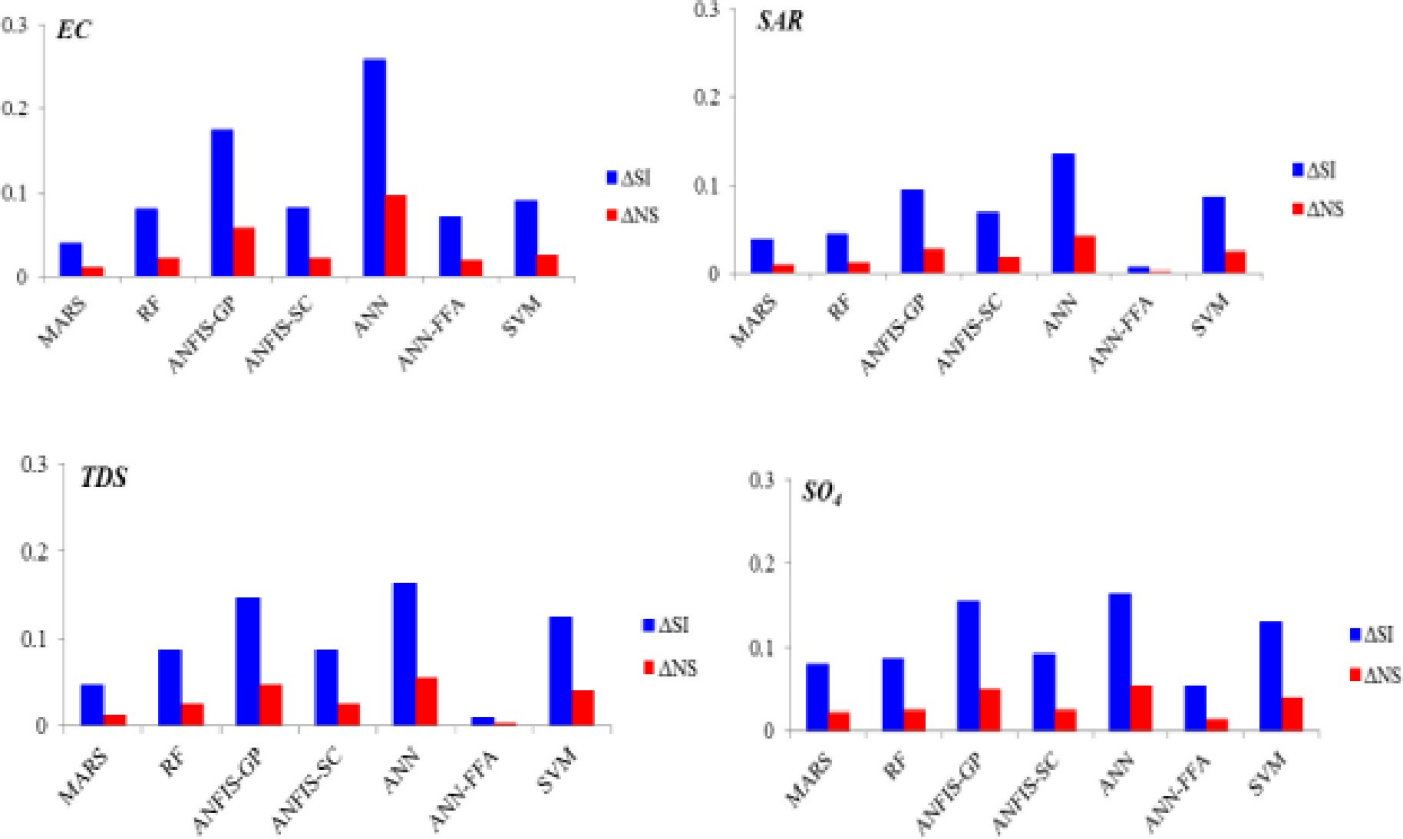
Improvements obtained through using SVM-FFA model by the second scenario in terms of *SI* reduction and NS increase.

The overall performance accuracy of *SAR* simulation models has been improved by using geographical coordinates of the wells. This might be due to the exclusion of other qualitative parameters that interact with *SAR* (and its governing vectors) from the input matrix and substituting by only geographical information, most likely to the mechanism followed by geo-statistical approaches. Fig 5 illustrates the improvements obtained through using SVM-FFA model by the second scenario in terms of *SI* reduction and *NS* increase for all studied parameters.

**Fig 6:**
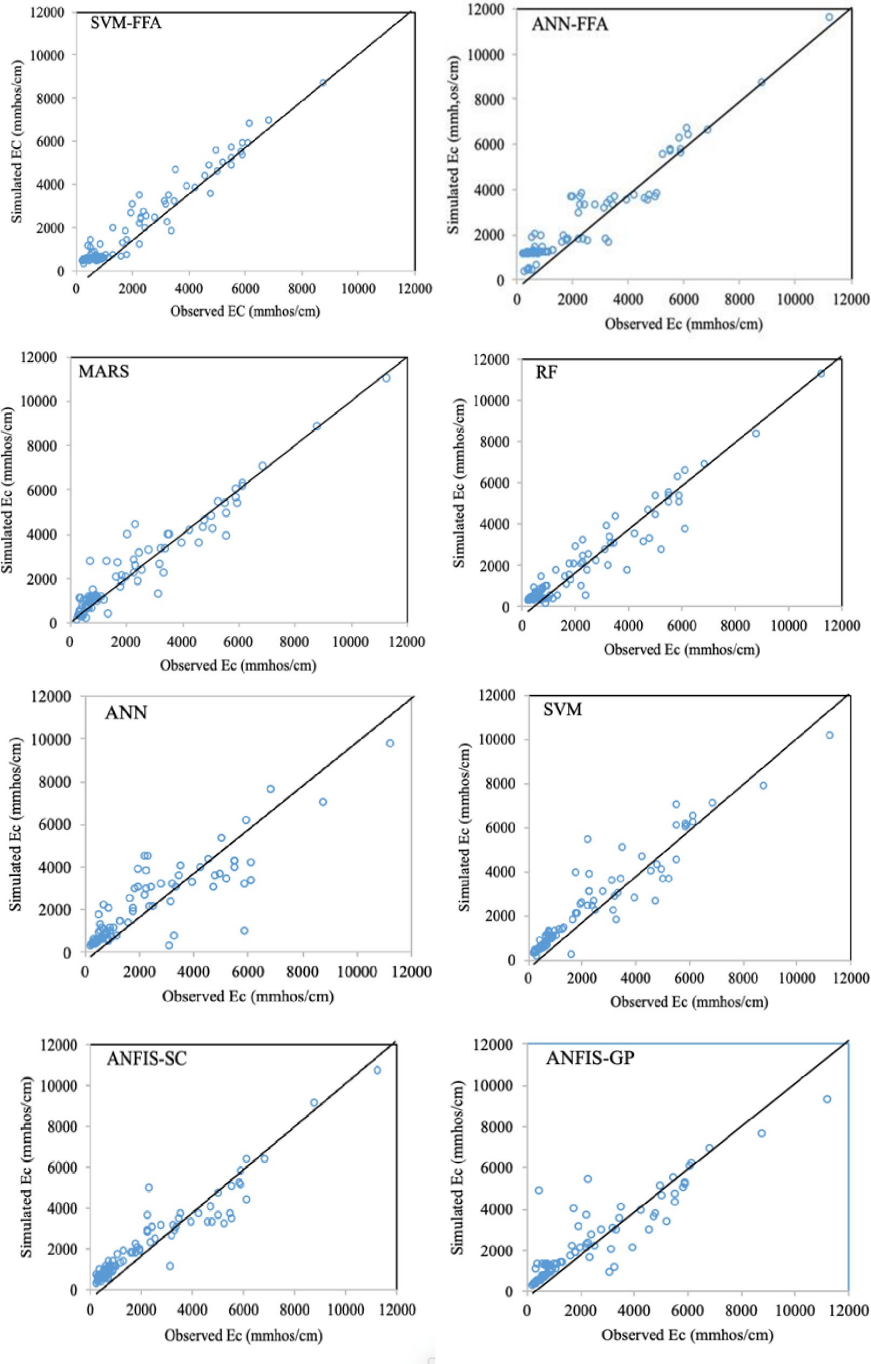
The scatterplots of predicted EC using the applied AI models.

**Fig 7:**
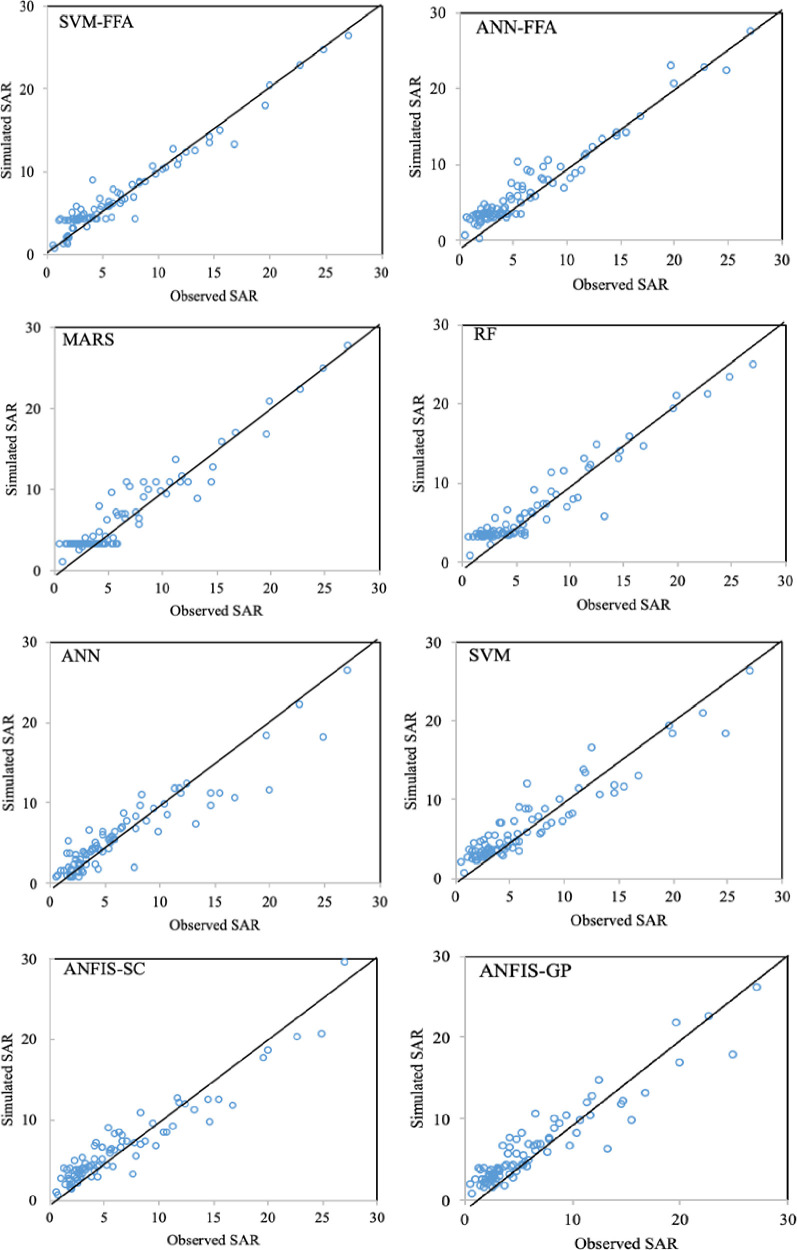
The scatterplots of the predicted SAR using the applied AI models.

**Fig 8:**
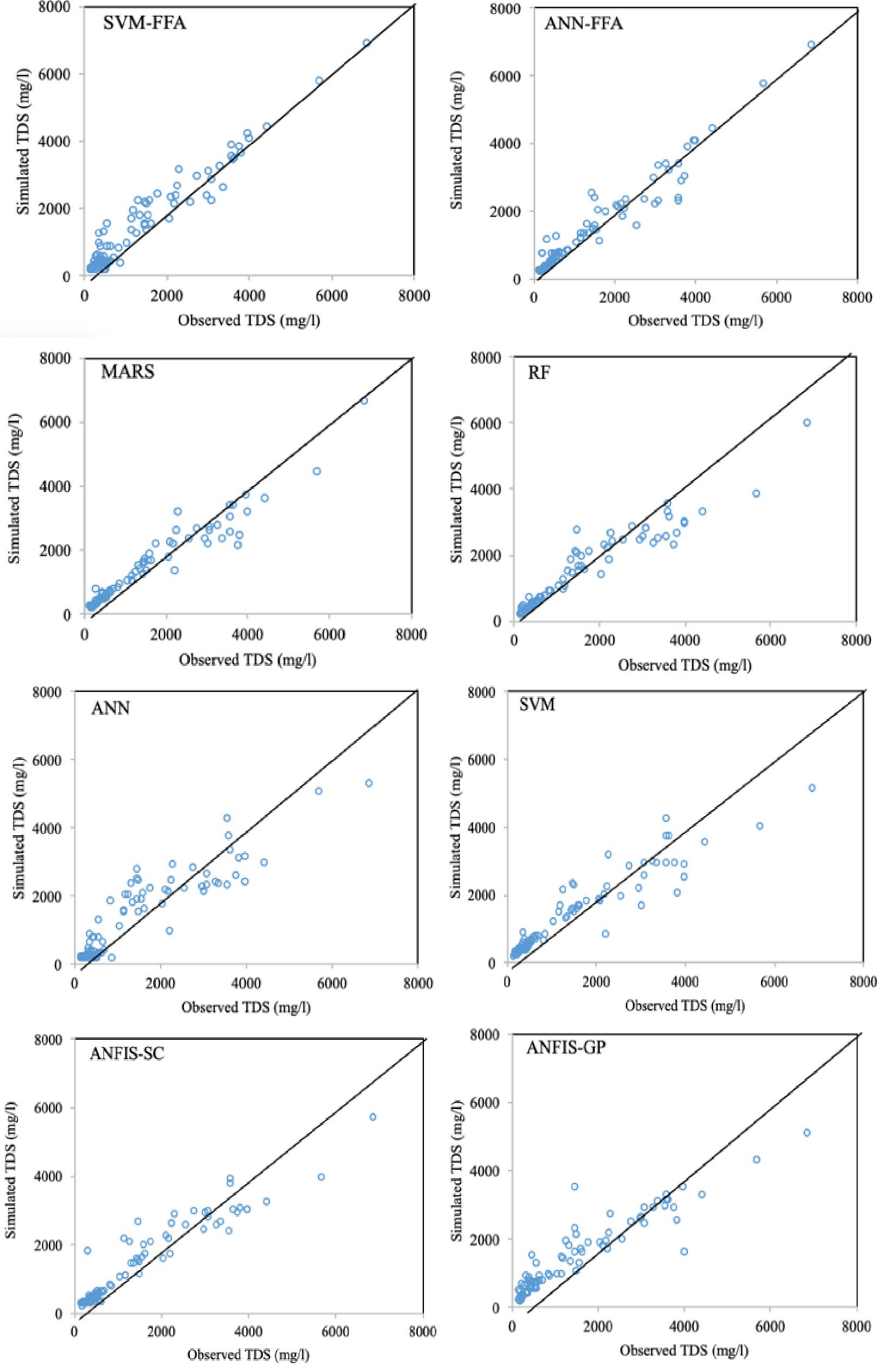
The scatterplots of the predicted TDS using the applied AI models.

**Fig 9:**
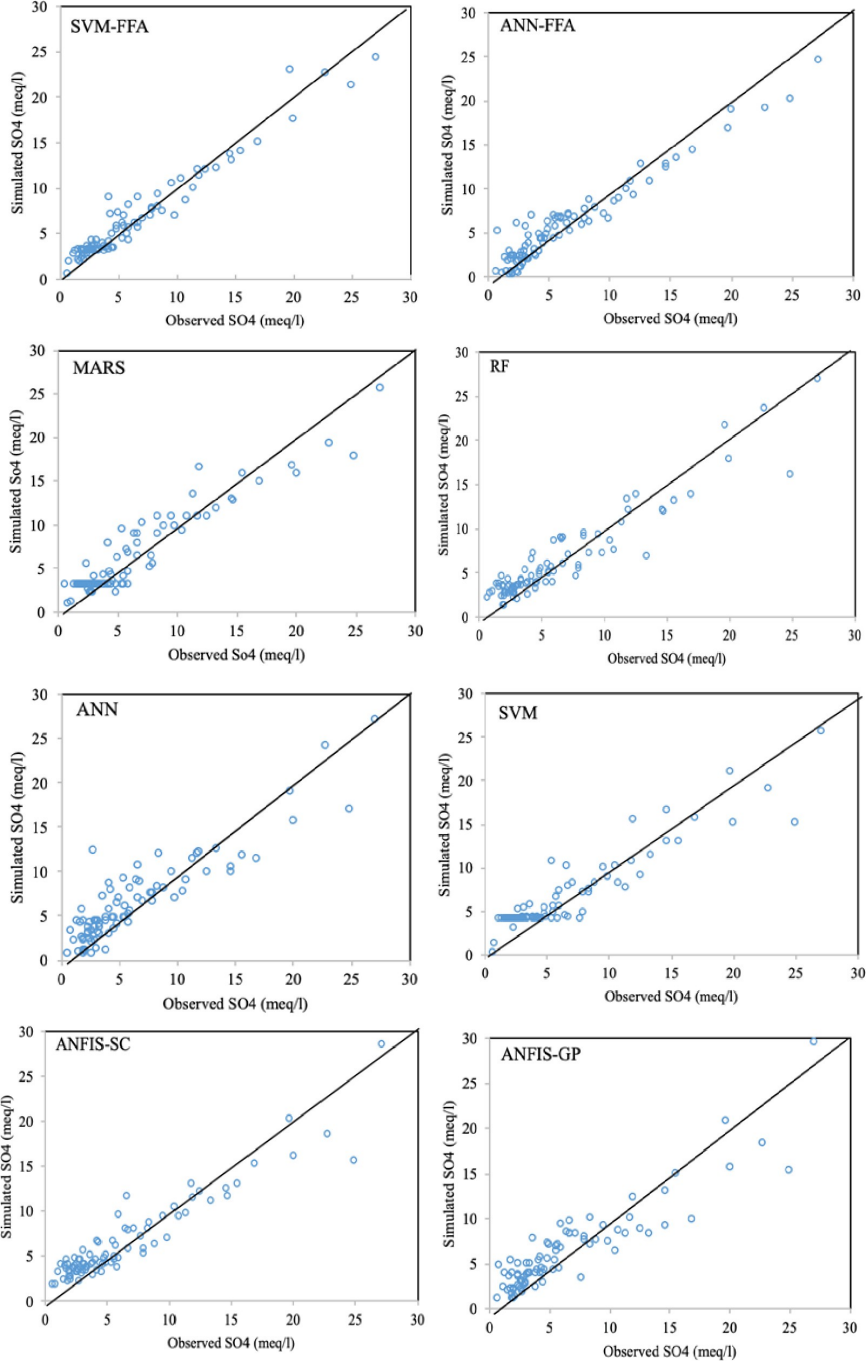
The scatterplots of the predicted SO_4_ using the applied AI models.

The maximum improvements belonged to EC simulation models by an average SI reduction of 11.3% and NS increase of 3.7%, while the lowest improvement values were observed for SAR modeling (ΔSI = 6.85% and ΔNS = 1.96%). In all the cases, the maximum SI and NS differences were observed between ANN and SVM-FFA models. For more informative evaluation of the predictive models on the water quality simulation, Figs 6–9 presented the observed and simulated values in the form of scatter plots of the studied parameters using the second scenario. The presented scatter plots reported an acceptable deviation from the ideal line of the ^o^45, this is confirming degree of the correlation between the observed and simulated dataset. However, in some cases, the high values of the SAR, TDS and SO_4_ failed to be simulated accurately using the standalone predictive models. Yet, the developed hybrid models revealed better degree of correlation as it can be observed for the SVM-FFA and ANN-FFA models.

The values presented here belong to the simulated/observed values of all test stages putted together. In case of EC simulations, the models have provided reasonable results for the lower EC magnitudes (good matches between the observed and simulated values), while some scatters are observed for higher values. The most scattered values are corresponded to the EC values between 2000–6000 mmhos/cm, while the values beyond 6000 gave better correspondence. Similar trend can be also observed for TDS modeling, too. For the SAR simulation models (Fig 7), all the applied models experienced difficulties with estimating lower values (especially SAR<5), which can be linked to lower sodium magnitudes in the soil that are leached through infiltration or deep percolation processes and make higher variations in their quantity. For the SO_4_ simulations, the lower values showed obvious scatters between the observed and simulated values, where similar values were obtained for different target values. As mentioned, the SVM-FFA and ANN–FFA provided the most accurate outcomes.

**Fig 10:**
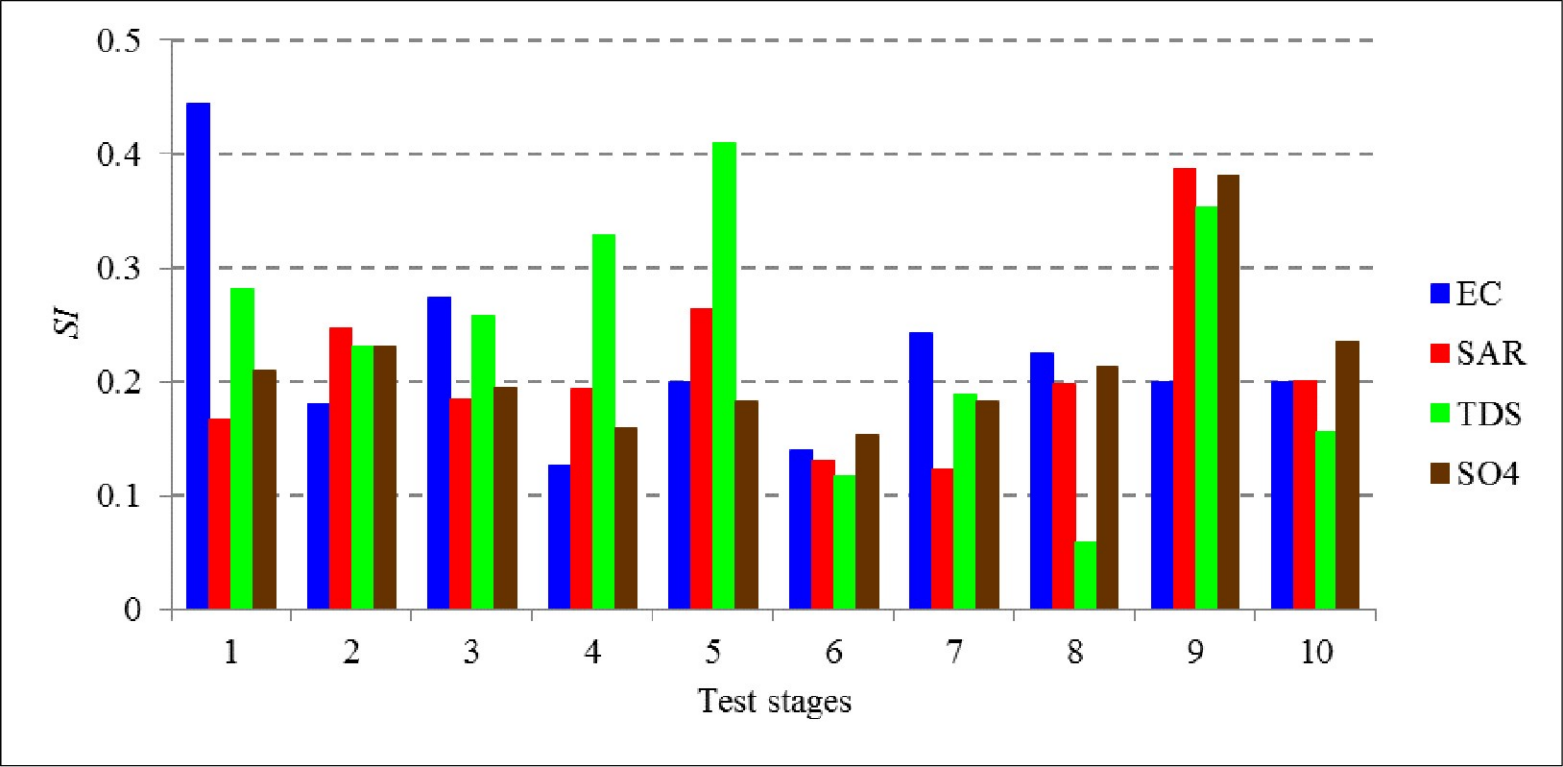
*SI* variations of the SVM-FFA model among the test stages (second scenario).

An additional analysis was also performed using the *SI* variations of SVM-FFA model “as a superior predictive model” among different test stages, as presented in Fig 10. Based on the results presented in Fig 10, *SI* values showed a clear variation among the different test stages for all variables. The maximum *SI* range (difference between its maximum and minimum values) was observed for *TDS (ΔSI* = 0.351) followed by EC, SAR and SO_4_ with 0.316, 0.263 and 0.227 *SI* ranges, respectively. This is clearly an evidence for the adopted k-fold testing data management strategy in assessing the soft computing models, without which, a deep through scanning of the models’ performances can’t be fulfilled.

## 4. Discussion

In Summary, it can be concluded that the applied models could simulate the spatial variations of the studied parameters with reasonable performance accuracies and meanwhile, SVM-FFA and ANN-FFA models outperformed the rest of the applied models with lower error magnitudes. Nonetheless, adoption of the robust k-fold testing strategy for assessing the applied models is a crucial task for avoiding the models’ over-fitting as well as getting more accurate insight about the performance accuracies of the applied models. The developed hybrid SVM-FFA and ANN-FFA models confirmed their applicability for simulating the groundwater quality owing to the robust learning process achieved through optimization algorithms used for the hyperparameters tuning of the model. This is an evidence with the established research over the literature. For instance, an artificial intelligence predictive model was established for groundwater quality parameters based on the hybridization of fuzzy c-means data clustering (FCM) with grid partition (GP), and ANN model hybridized using particle swarm optimization (PSO) [72]. The proposed hybrid FCM-GP reported better results in comparison with the ANN-PSO model. The application of computer aided models such as the one proposed one in the current research can be sufficiently applicable for geo-science engineering monitoring and control. Decision makers and environmental engineers could substantially benefit from knowing the future pattern of the groundwater quality in which an appropriate determination can take place for groundwater usage and sustainability.

For future research direction, the data, models and input parameters uncertainties could be further analyzed and discussed [73, 74]. Finally, global comparison of both the adopted scenarios revealed that, although considerable differences were not observed between the scenarios, the second scenario could provide promising outcomes in simulating groundwater quality parameters. As the second scenario considers only the geographical information as input parameters, this performance accuracy is exclusively important in practical problems, where the available data are not sufficient or reliable. One may tackle this issue with criticizing the comparative superiority of one scenario over the other one, but it should be noted that, the goal with this research was assessing the capabilities of soft computing techniques in estimating groundwater quality parameters through using limited easily accessible data, so information around the reasons of any scenario’s superiority is not discussible/accessible there. Just the point that might be discussed would be the ability of the applied models in simulating the spatial variations of those parameters through using the geographical data.

## 5. Conclusion

A modeling study was performed here by using eight soft computing approaches for spatial modeling of four important groundwater quality parameters, viz. EC, SAR, TDS and SO_4_ using data from 90 observation wells in Northwest Iran. Two modeling protocols were followed for simulation of the target variables: first, other quality parameters were used to estimates the targets and second, only the geographical information (coordinates) was introduced as inputs. Based on the obtained results, all applied models could simulate the target values with acceptable accuracy although the coupled SVM-FFA surpassed the others with the highest performance accuracy. Nevertheless, the models showed good ability to simulate the target values when relying on geographical inputs. Finally, the importance of adopting a k-fold testing strategy was confirmed to make a through scanning of the applied models, because considerable fluctuations of the models’ performance accuracy were observed with respect to the selected test stages. The outcomes of the present paper encourage making further comparative studies among soft computing approaches using data from other regions with different climatic contexts and data availability to strengthen the obtained conclusions.
